# A screen for novel hepatitis C virus RdRp inhibitor identifies a broad-spectrum antiviral compound

**DOI:** 10.1038/s41598-017-04449-3

**Published:** 2017-07-19

**Authors:** Abhilasha Madhvi, Smita Hingane, Rajpal Srivastav, Nishant Joshi, Chandru Subramani, Rajagopalan Muthumohan, Renu Khasa, Shweta Varshney, Manjula Kalia, Sudhanshu Vrati, Milan Surjit, C. T. Ranjith-Kumar

**Affiliations:** 1 Vaccine and Infectious Disease Research Center, Translational Health Science and Technology Institute, Faridabad, India; 2 Shiv Nadar University, Gautam Buddha Nagar, Uttar Pradesh, India; 3 Regional Centre for Biotechnology, Faridabad, India

## Abstract

Hepatitis C virus (HCV) is a global pathogen and infects more than 185 million individuals worldwide. Although recent development of direct acting antivirals (DAA) has shown promise in HCV therapy, there is an urgent need for the development of more affordable treatment options. We initiated this study to identify novel inhibitors of HCV through screening of compounds from the National Cancer Institute (NCI) diversity dataset. Using cell-based assays, we identified NSC-320218 as a potent inhibitor against HCV with an EC_50_ of 2.5 μM and CC_50_ of 75 μM. The compound inhibited RNA dependent RNA polymerase (RdRp) activity of all six major HCV genotypes indicating a pan-genotypic effect. Limited structure-function analysis suggested that the entire molecule is necessary for the observed antiviral activity. However, the compound failed to inhibit HCV NS5B activity *in vitro*, suggesting that it may not be directly acting on the NS5B protein but could be interacting with a host protein. Importantly, the antiviral compound also inhibited dengue virus and hepatitis E virus replication in hepatocytes. Thus, our study has identified a broad-spectrum antiviral therapeutic agent against multiple viral infections.

## Introduction

Hepatitis C virus is one of the leading causes of acute and chronic liver diseases. Approximately 4 million new HCV infections occur every year with a fatality rate of more than 50,000 each year. HCV infection is the cause for more than 50% of hepatocellular carcinoma, which is the fastest growing cause of cancer-associated deaths in the United States^1, 2^. There are no vaccines available to combat HCV and its treatment is highly dependent on antiviral drugs. Prior to year 2013, pegylated interferons and ribavirin was standard of care for HCV infections, which is now being replaced by direct acting antivirals (DAA)^3–5^.

There are six major genotypes of HCV and response to DAA treatment can vary according to the genotype. HCV genotype 3 accounts for 40% of all HCV infections in Asia and is predominant in some European countries such as Norway, Denmark, Finland and United Kingdom^6, 7^. Moreover, the risk of developing severe liver injuries such as cirrhosis and hepatocellular carcinoma is much higher with genotype 3^8^. While introduction of DAAs has revolutionized therapy against genotype 1 virus, it displayed inferior responses against HCV genotype 3^9^. Furthermore, the costs of the DAAs are so high that in many countries few patients are able to afford them. With more than 85% of the chronic HCV patients residing in low- and middle-income countries, this high price tag is the most significant barrier for availing treatment in these countries^10, 11^. Hence, development of new affordable treatments is necessary to ensure access to patients around the world.

Apart from HCV, there are a multitude of emerging and re-emerging RNA viruses with limited antiviral therapy available. Dengue virus (DENV) and hepatitis E virus (HEV) infections are a couple of such major global health problems. About 40% of the world population lives in the areas having the risk of dengue infection. Astoundingly, more than 3.9 billion people living in over 128 countries are under the threat of dengue infection^12^. HEV causes self-limiting hepatitis with an estimated 20 million infections and 3.3 million acute cases annually worldwide. It causes chronic infection in organ transplant or immunocompromised individuals and a 30% mortality rate is seen in pregnant women in developing countries^13, 14^.

Recently, a tetravalent vaccine has been approved for dengue and a recombinant subunit vaccine for HEV was registered in China^15, 16^. However, the efficacy of dengue vaccine is very modest in seronegative individuals and the efficacy of the HEV vaccine in immunocompromised individuals is not known. Development of dengue vaccines is facing difficulties because of the antibody-dependent enhancement (ADE) phenomena^17^. Non-neutralizing antibodies produced during infection of one DENV serotype contributes to the enhancement of disease severity upon infection by one of the other three serotypes^18^. Therefore, there is an urgent requirement of antiviral drugs to treat or prevent these viral infections.

In this study we sought to identify antivirals, through screening a library of 1363 biologically active small molecules belonging to the diversity set II of National Cancer Institute (NCI) Developmental Therapeutic Program (DTP). Using HCV genotype 3a NS5B cell based assay and replicon assay, we identified one antiviral compound, NSC-320218 [IUPAC name 1-(9-ethylcarbazol-3-yl)-3-(2-methyl-4-nitrophenyl)urea], referred to as 66E2. It displayed pan-genotypic inhibition of HCV as well as DENV and HEV replication in hepatocytes. Further analysis showed that the broad-spectrum inhibitor, 66E2 had no cytotoxic effects up till 20 μM and could be acting through host factor(s). Thus our study has identified a promising anti-HCV compound that exhibits a broad-spectrum antiviral effect.

## Results

### Screening of compound library

**Figure 1:**
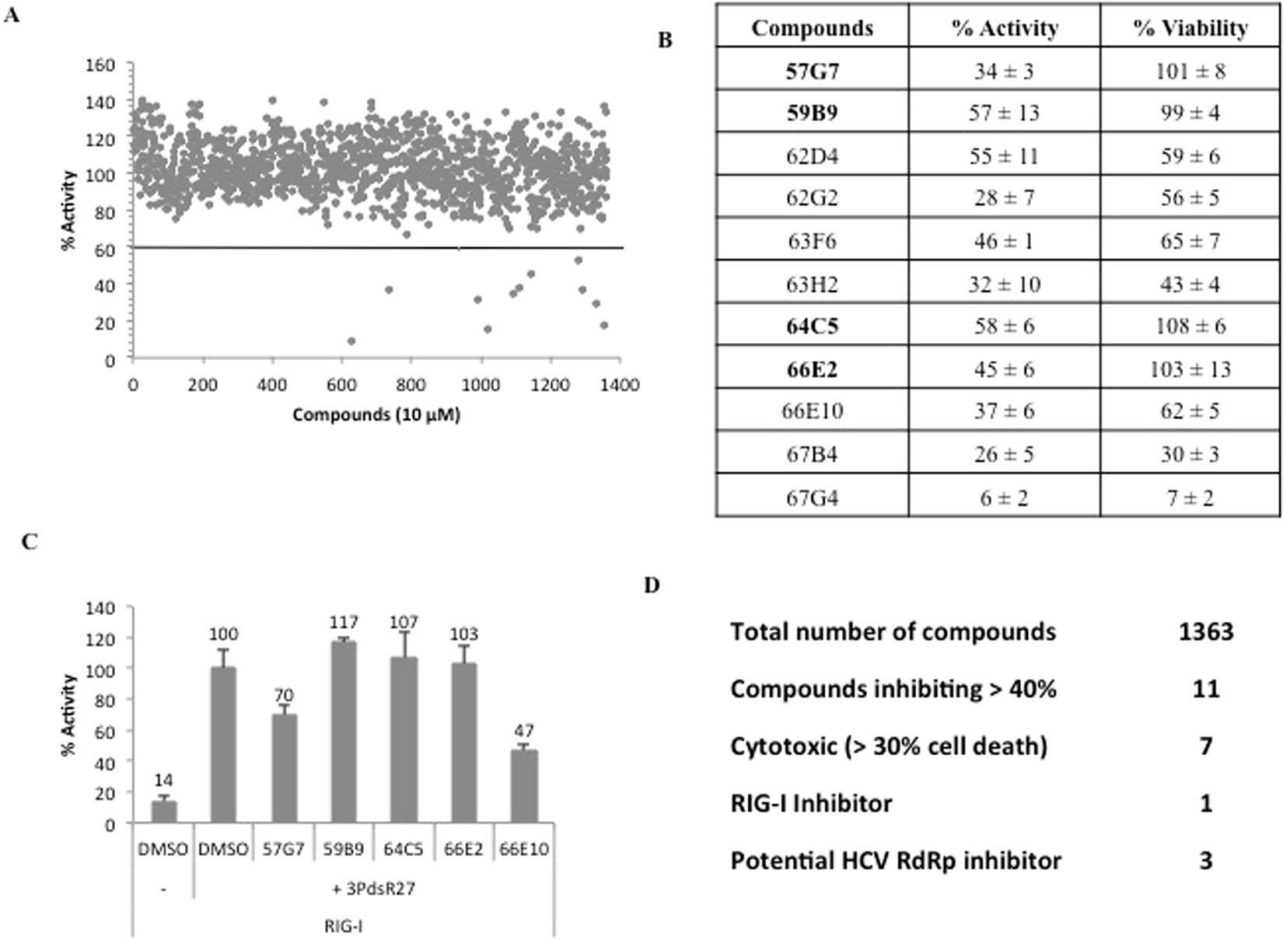
Cell-based screening. (**A**) Each circle represents the mean % of the ratio of firefly luciferase to *Renilla* luciferase (referred to as % Activity) for a given compound tested at 10 μM in duplicate for 48 h. 11 compounds showed values less than 60% (horizontal line). (**B**) The same 11 compounds were retested in the cell-based assay in triplicates and their cytotoxicity analyzed using WST assay. The results are representative of three independent assays. The means and standard deviations of each result are shown. The values correspond to the ratio of firefly luciferase to *Renilla* luciferase (% activity) and % of live cells (% viability) upon treatment with respective compounds at 10 μM. The compounds in bold are the ones that inhibited NS5B activity without exhibiting any cell toxicity. (**C**) RIG-I assay to test the specificity of the compounds. Compounds that showed more than 40% inhibition without any cytotoxicity in B were tested along with the cytotoxic compound 66E10. RIG-I was induced with a 27 bp triphosphorylated dsRNA, 3P dsR27. The % activity is plotted against each compound with DMSO as control. % Mean is shown above the bars and the error bars are standard deviations. The assays were performed in triplicates and results presented are representative of three independent assays. (**D**) Table summarizing the data from (**A**–**C**).

We had earlier established a cell-based assay for hepatitis C virus (HCV) genotype 1b RNA dependent RNA polymerase (RdRp, Fig. S1A) and showed that the assay could be used to identify inhibitors^19^. Since previously identified HCV inhibitors have performed less efficiently against genotype 3, we optimized the cell-based assay with full-length genotype 3a NS5B (Fig. S1B). Similar to HCV-1b NS5B, activity of the HCV-3a NS5B cell-based assay was dependent on the catalytically active NS5B and a functional RIG-I (Fig. S1B)^19^. Employing this assay we screened a library of 1363 biologically active small molecule compounds belonging to the diversity set II of NCI DTP to identify novel inhibitor of HCV-3a RdRp (Fig. 1A). All the compounds were tested at 10 μM. The criterion for an active compound was the one that exhibited more than 40% reduction in activity compared to the DMSO treated control (set to 100%). Eleven molecules inhibited the activity by more than 40%. After retesting the inhibitory potential of these compounds using the cell-based assay, we tested their cytotoxicity using WST-1 assay, in parallel (Fig. 1B). Seven of the identified compounds reduced cell viability by more than 30% and hence were discounted from further characterization. The remaining four compounds (57G7, 59B9, 64C5 and 66E2) that appeared to inhibit RdRp activity without exhibiting cytotoxicity were characterized further (highlighted in Fig. 1B).

Since our cell-based assay uses RIG-I signaling pathway (Fig. S1A) and ref. 19, we evaluated if any of the identified compounds inhibited RIG-I pathway rather than HCV NS5B. To validate the specificity of these compounds, we tested them on RIG-I signaling assay using a triphosphorylated dsRNA as RIG-I agonist (Fig. 1C). Out of the four identified inhibitors, compound 57G7 inhibited RIG-I signaling, suggesting that it may not be a 3a NS5B specific inhibitor. 66E10, which showed significant cytotoxicity, also inhibited RIG-I signaling (Fig. 1B). Thus, we obtained 3 potential inhibitors (59B9, 64C5 and 66E2) of HCV-3a NS5B activity (summarized in Fig. 1D).

### Effect on HCV genotype 3a replicon

**Figure 2:**
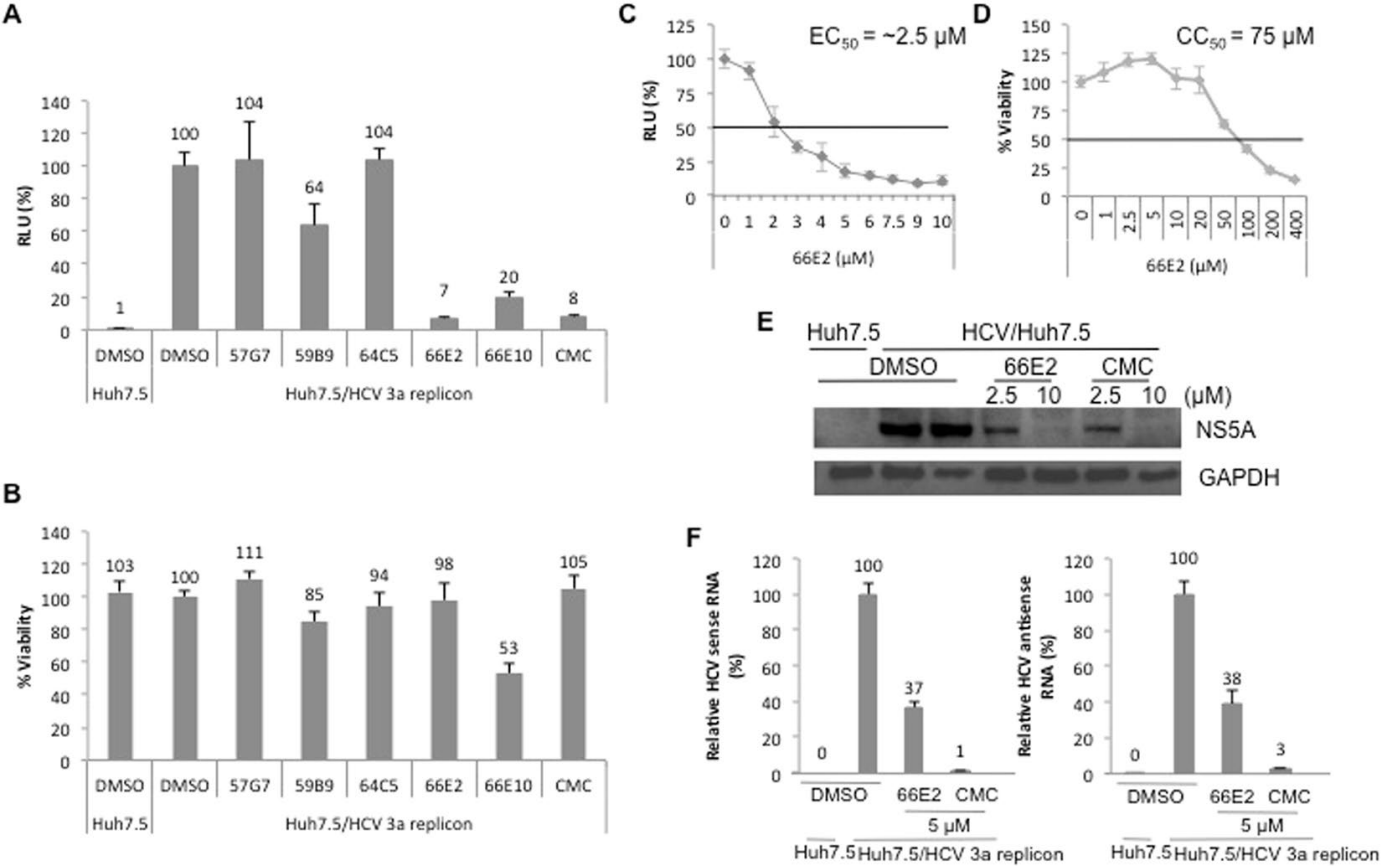
Assays with HCV genotype 3a replicon. (**A**) G418 resistant HCV-3a replicon expressing Huh7.5 cells were treated with indicated compounds for 48 h and the firefly luciferase activity was plotted as relative luciferase units (RLU). DMSO treated HCV-3a replicon expressing Huh 7.5 cells was taken as 100%. (**B**) The toxicity of these compounds in the replicon expressing cells was measured using WST-1 assay reagent. The values are depicted as percentages with the DMSO treated cells taken as 100%. (**C**) The replicon expressing cells were treated with varying concentrations of 66E2 and relative luciferase unit is plotted against the concentration of 66E2. EC_50_ is the compound concentration that inhibits 50% of viral replication (RLU). (**D**) Huh7.5 cells were treated with indicated concentrations of 66E2 and cytotoxicity determined using WST-1 assay reagent. The values are plotted as percentages with the DMSO treated cells taken as 100%. CC_50_ is the compound concentration that produces 50% of cytotoxicity. (**E**) Western blot analysis. Replicon expressing Huh7.5 cells were treated with different concentrations of 66E2 and CMC for 48 hours and expression of NS5A monitored using anti NS5A antibody. Expression of GAPDH was determined for loading control. (**F**) Reverse-transcriptase quantitative PCR analysis was performed to determine the levels of positive and negative sense HCV-3a RNA upon treatment with 66E2 and CMC at 5 μM for 48 h. The % mean is shown above the bars and the error bars are standard deviations. All the assays in the figure were performed in triplicates and results presented are representative of at least three independent assays.

In addition to RdRp, the HCV replicase complex consists of other viral encoded non-structural proteins (NS3-NS5B) as well as host proteins. In order to evaluate the ability of the selected compounds to inhibit NS5B when present as part of the replicase complex, we tested their inhibitory capacity in Huh7.5 cells transfected with HCV genotype 3a replicon RNA^20^ (Fig. 2A). The HCV-3a replicon expresses a chimeric fusion protein of firefly luciferase and neomycin phosphotransferase and therefore could be selected using G418. The G418 resistant colonies show luciferase activity in proportion to the HCV RNA replication^20^. The G418-resistant replicon expressing Huh7.5 cells were treated with the potential HCV RdRp inhibitors along with a known inhibitor, 2′-C-methylcytidine (CMC)^21^, (Fig. 2A). Interestingly, similar to CMC, only 66E2 (at 10 μM) inhibited HCV-3a replicon without any effect on cell viability in the replicon expressing Huh7.5 cells (Fig. 2A and B). 57G7 did not show any inhibition further confirming that it may be a RIG-I antagonist. As expected, 66E10 again showed significant cytotoxicity (Fig. 2B). Compounds 59B9 and 64C5 were unable to show any significant inhibition suggesting that while they could inhibit NS5B in the cell based assay, they were unable to access their target in the replicase complex. To further confirm this, we tested 59B9 and 64C5 along with 66E2 at 20 and 50 μM (Fig. S2). While 66E2 inhibited HCV replicon almost completely, 64C5 and 59B9 inhibited 43% and 67% respectively at 50 μM (Fig. S2A). However, 66E2 and 59B9 showed significant cytotoxicity at 50 μM concentration (Fig. S2B). Since very high concentrations of 64C5 and 59B9 were necessary to inhibit HCV replicon, these compounds were not considered further. Thus, 66E2 inhibited HCV-3a NS5B when present alone or in replicase complex with no apparent cell toxicity.

Titration of 66E2 revealed that its EC_50_ is approximately 2.5 μM (Fig. 2C) and CC_50_ is around 75 μM (Fig. 2D). To further confirm the antiviral effect of 66E2, we monitored the expression of nonstructural protein 5A (NS5A) by western analyses. As expected, 66E2 inhibited NS5A expression in replicon expressing cells similar to CMC (Fig. 2E). Lastly, quantitative PCR analyses were performed to measure both positive and negative sense HCV-3a replicon RNA (Fig. 2F). A 60% reduction in both positive and negative sense viral RNA was observed at 5 μM of 66E2, further confirming the antiviral properties of 66E2.

### Structure-activity relationship

**Figure 3:**
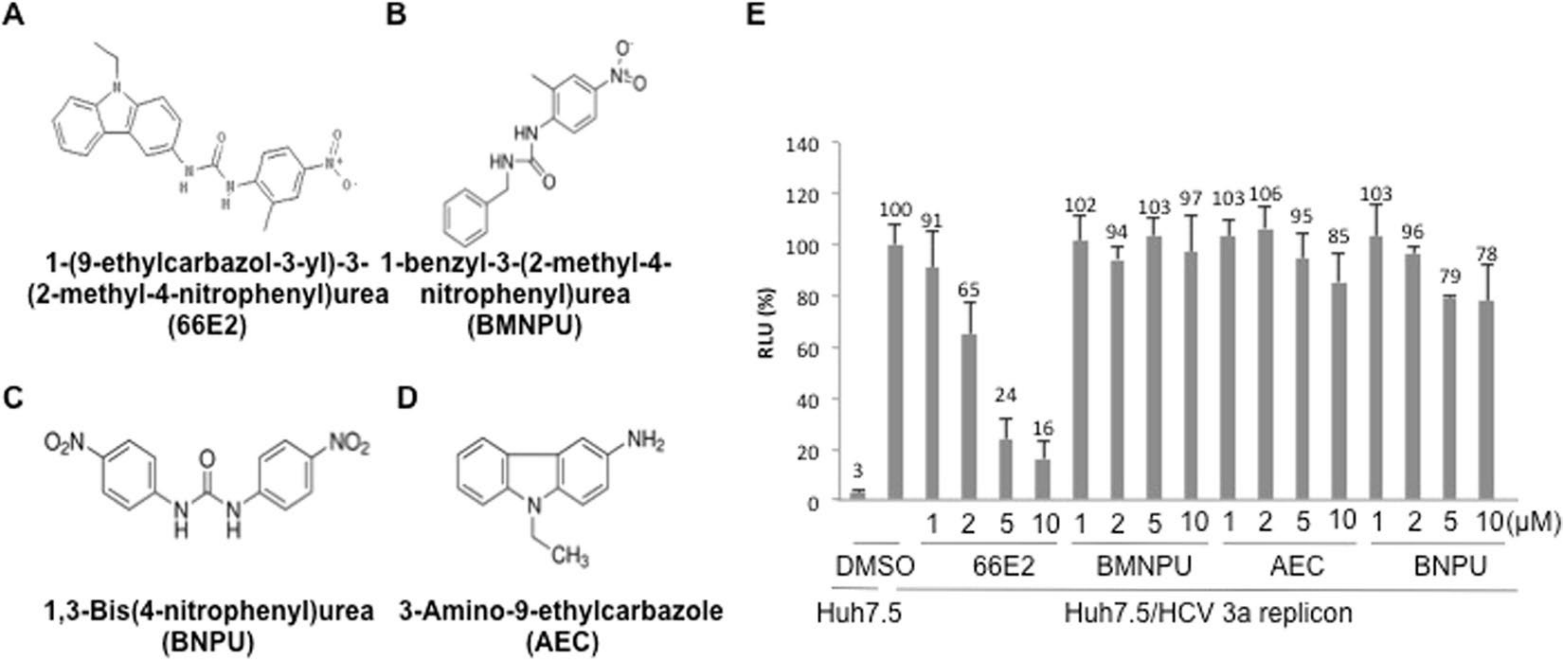
Structure Activity Relationship Analysis. (**A**–**D**) Structure of 66E2 and its derivatives are depicted. (**E**) G418 resistant HCV-3a replicon expressing Huh7.5 cells were treated with varying concentrations of these compounds. Levels of RNA replication was monitored using the firefly reporter and plotted as % RLU as in Fig. 2A. The assays were performed in triplicates and results presented are representative of three independent assays. The % mean is shown above the bars and the error bars are standard deviations.

The IUPAC name of 66E2 is 1-(9-ethylcarbazol-3-yl)-3-(2-methyl-4-nitrophenyl)urea and its structure is represented in the Fig. 3A. A preliminary structure-activity relationship was explored with different moieties of 66E2 i.e the (4-nitrophenyl)urea and the ethylcarbozole group (Fig. 3A–D). The potency of (4-nitrophenyl)urea was tested using two compounds 1-benzyl-3-(2-methyl-4-nitrophenyl)urea (BMNPU) and 1,3-Bis(4-nitrophenyl)urea (BNPU) (Fig. 3B and C) and the role of ethylcarbozole group was analyzed using 3-Amino-9-ethylcarbazole (AEC, Fig. 3D). Interestingly, while 66E2 inhibited HCV-3a replication, none of its derivatives were able to show any significant inhibition (Fig. 3E). Our limited structure-activity relationship analysis suggests that for potent inhibition of HCV both the carbozole group and the nitrophenyl urea group are essential.

### 66E2 inhibits RdRp activity from all major HCV genotypes

**Figure 4:**
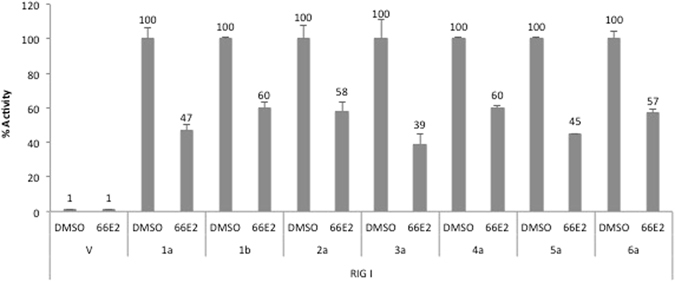
Pangenotypic effect of 66E2. Cell-based assay was performed with RdRps from all six major genotypes of HCV, along with vector (V) as control, in the presence of 10 μM 66E2. DMSO was used as a control. The assays were performed in triplicates and results presented are representative of three independent assays. The % mean is shown above the bars and the error bars are standard deviations.

Next, we explored whether 66E2 is specific to genotype 3a or it could inhibit other genotypes of HCV. Cell-based assay for RdRps from genotypes 1a, 1b, 2a, 3a, 4a, 5a and 6a were performed in the presence of 66E2 (at 10 μM) along with DMSO as control (Fig. 4). 66E2 was capable of inhibiting the activity of RdRps of all HCV genotypes ranging from 40–60% suggesting that it is a pan-genotype inhibitor of HCV.

### 66E2 does not inhibit recombinant HCV RdRp *in vitro*

**Figure 5:**
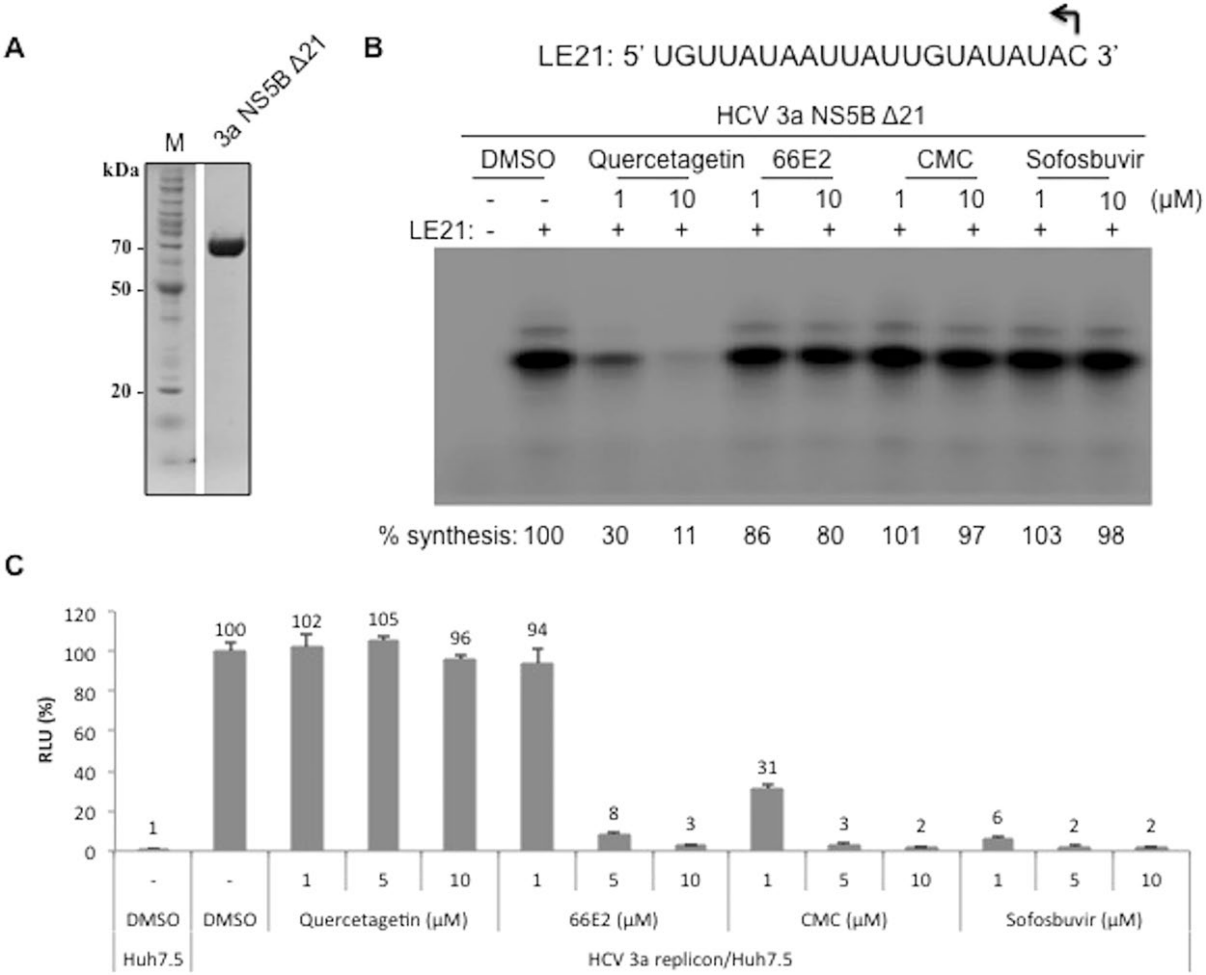
66E2 does not inhibit HCV RdRp *in vitro*. (**A**) Coomassie stained SDS-PAGE depicting HCV RdRp lacking C-terminal 21 amino acids (3a NS5B Δ21) that was expressed in *E*. *coli* and purified to near homogeneity (lane 2). Lane 1, Molecular weight marker. (**B**) RNA synthesis *in vitro* by the 3a NS5B Δ21 in the presence of 66E2 and other known HCV inhibitors. The sequence of the template (LE21) used is given on the top. The amount of the 21-nt de novo initiated product synthesized was quantified and given as percent synthesis relative to the sample treated with only DMSO. The results presented are representative of three independent assays. (**C**) G418 resistant HCV-3a replicon expressing Huh7.5 cells were treated with varying concentrations of inhibitors. RNA replication is presented as % RLU. Reaction treated with DMSO was considered as 100%. The assays were performed in triplicates and results presented are representative of three independent assays. The % mean is shown above the bars and the error bars are standard deviations.

One of the ways that 66E2 inhibits HCV could be by directly acting on the viral RdRp. To test this hypothesis, we expressed and purified HCV genotype 3a NS5BΔ21 from *E*. *coli* (Fig. 5A) and performed an *in vitro* RdRp assay using a 21-nucleotide RNA, LE21, as template (Fig. 5B)^22^. The flavonol, quercetagetin, a known inhibitor of HCV NS5B was used as positive control^23^. While quercetagetin showed potent inhibition, 66E2 failed to inhibit recombinant HCV-3a NS5BΔ21 mediated RNA synthesis (Fig. 5B). This suggests that 66E2 may not be directly acting on the viral polymerase. In addition, two known prodrugs of HCV, CMC and sofosbuvir also failed to inhibit recombinant HCV NS5BΔ21 (Fig. 5B). Being prodrugs, they undergo metabolic changes inside the cells to form the active compounds. To confirm this, we added these compounds to HCV-3a replicon expressing Huh7.5 cells (Fig. 5C). Surprisingly, quercetagetin was unable to inhibit HCV-3a replicon in Huh7.5 cells while CMC and sofosbuvir as well as 66E2 inhibited HCV-3a replicon as expected (Fig. 5C). Taken together these data suggest that 66E2 is either undergoing a metabolic change inside the cells to convert into an active form or is interacting with a host protein necessary for HCV replication.

### 66E2 also inhibits dengue and hepatitis E virus replication

**Figure 6:**
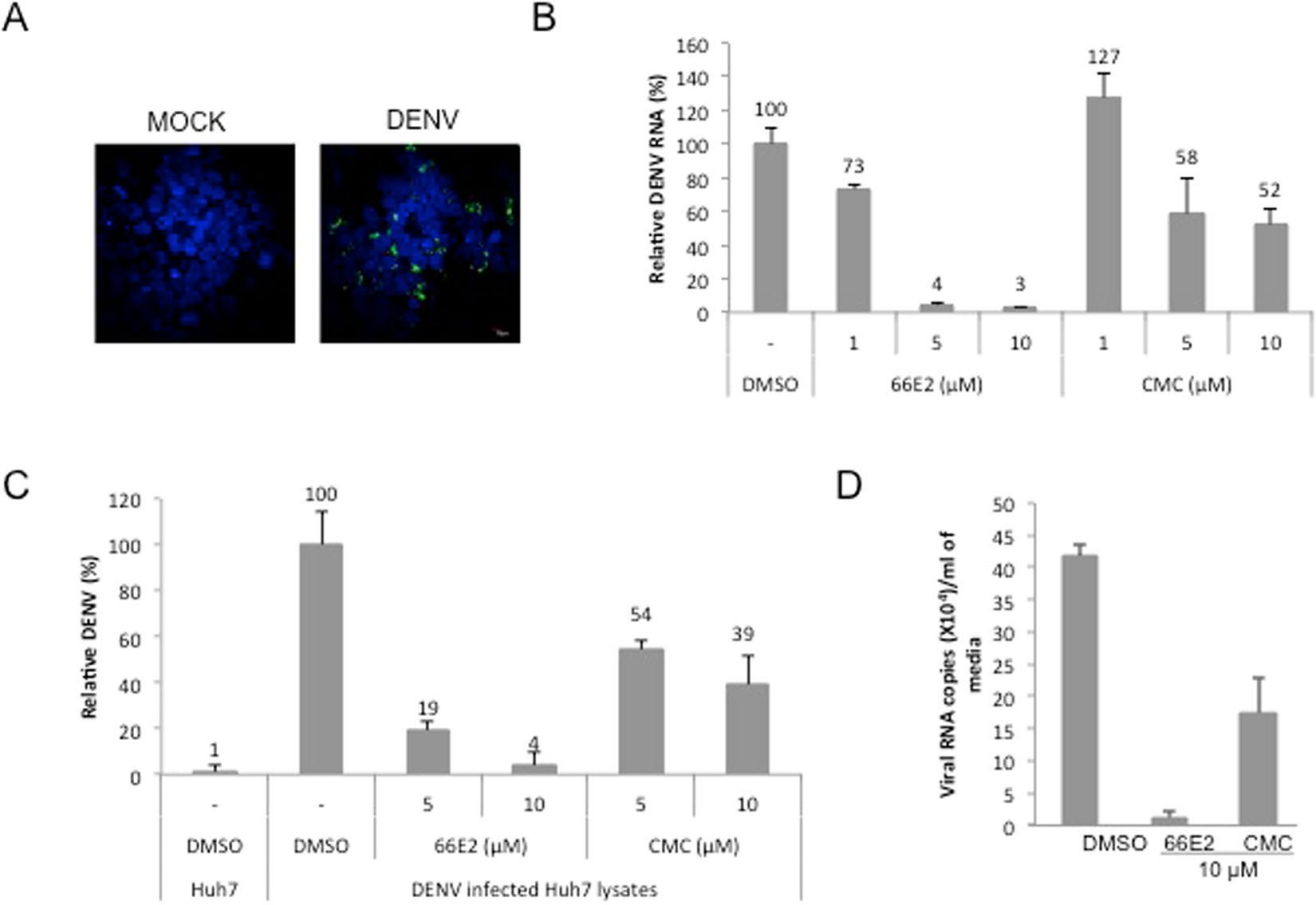
Effect of 66E2 on dengue virus infection. (**A**) Immunofluorescence microscopy showing DENV infection of Huh7 cells. Huh7 cells were infected with DENV-2 at 1MOI. At 48 hpi cells were fixed and stained with anti-dengue virus antibody (ab9202) and Alexa 488 anti-mouse antibody (green). Nuclei were stained with DAPI (blue). About 40% of the cells are infected. (**B**) Huh7 cells were infected with DENV-2 virus (at 1 MOI) in the presence of indicated concentrations of 66E2 and CMC. At 48 hpi total RNA was extracted and reverse-transcription quantitative PCR analysis was performed to determine the levels of DENV replication. Results are expressed as the percentage relative to DMSO treated control. (**C**) ELISA to detect levels of DENV core protein produced inside Huh7 cells infected with DENV-2 virus in the presence of 66E2 and CMC. Uninfected Huh7 cells were used as control. Results are expressed as relative percentages to DMSO treated control. For both (**B** and **C**), results are representative of three independent assays performed in triplicates. The % mean is shown above the bars and the error bars are standard deviations. (**D**) Huh7 cells were infected with DENV-2 at 1 MOI. At 48 hpi, total RNA was extracted from the media and viral RNA copy number/ml of the media in the presence of 10 μM of 66E2 and CMC was estimated and plotted against the compounds used. DMSO treated cells were used as control.

In order to establish whether 66E2 specifically inhibited HCV, we explored its effect on the replication of two other RNA viruses such as DENV and HEV. First, Huh7 cells were infected with DENV at 1 MOI and immunofluorescence assay with anti-dengue antibody indicated close to 40% infection at 48 hpi (Fig. 6A). We then tested the compound against dengue virus type 2 (DENV-2) by infecting Huh7 cells at 1 MOI followed by the addition of 66E2. DMSO and CMC were used as the negative and positive controls, respectively^24^. At 48 hpi, 66E2 inhibited dengue replication by 90% at 5 μM whereas CMC inhibited only around 50% as monitored by quantitative PCR (Fig. 6B). To confirm this observation, we performed ELISA to detect the expression of DENV core protein in the presence and absence of 66E2 (Fig. 6C). Similar to the quantitative PCR data, 66E2 was more potent than CMC and showed an almost complete inhibition of DENV core protein expression at 5 and 10 μM of the compound. Lastly, estimation of the copy number of DENV RNA released into the medium in the presence and absence of 10 μM 66E2 and CMC further confirmed the superior inhibitory effect of 66E2 compared to CMC (Fig. 6D).

**Figure 7:**
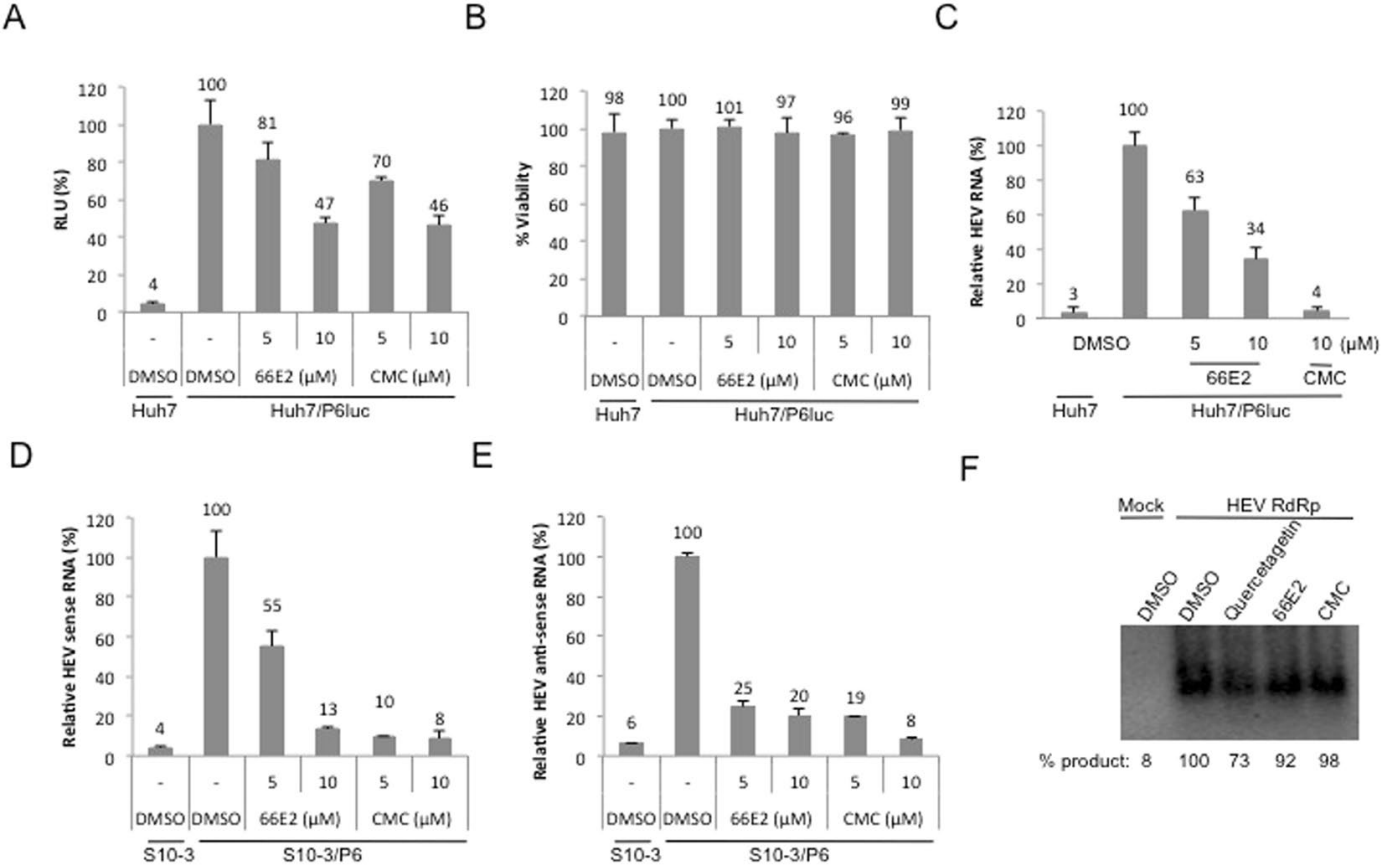
66E2 inhibits hepatitis E virus replication. (**A**) P6Luc expressing Huh7 cells were treated 66E2 and CMC. At 48 h post-infection guassia luciferase secreted into the medium was monitored and plotted as % RLU. DMSO treated Huh7 cells were used as control. (**B**) Cytotoxicity of the compounds was tested and the values are depicted as percentages with the DMSO treated cells taken as 100%. (**C**) Reverse-transcriptase quantitative PCR analysis was performed to determine the levels of HEV replication in the p6luc transfected Huh7 cells in the presence of 66E2 and CMC. Results are expressed as the percentage relative to DMSO treated control. (**D** and **E**) S10-3 cells were infected with p6 virus in the presence of 66E2 and CMC along with DMSO as vehicle control. After 48 h, total RNA was extracted and reverse-transcriptase quantitative PCR analysis was performed to determine the levels of positive (**D**) and negative sense (**E**) p6 RNA. Results are expressed as % relative to DMSO treated control and are representative of two independent assays. All the assays in this figure were performed in triplicates and results presented are representative of three independent assays. (**F**) RdRp assay using purified HEV RdRp. RNA harboring 130 bases from 5′ end and 210 bases from 3′ end of HEV genome was used as a template. The amount of product formed is quantified and given below as percent synthesis relative to the sample treated with DMSO. The data presented are representative of three independent experiments.

Next, we analyzed the effect of 66E2 on hepatitis E virus (HEV genotype 3) replicon, p6/luc system. HEV p6/luc replicon generates a secretable gaussia luciferase expressed from subgenomic RNA^25^. The presence of luciferase expressed via subgenomic RNA indicates that viral replication has occurred. The p6/luc reporter system was optimized (Fig. S3) and employed to test the effect of 66E2. CMC was earlier reported to inhibit HEV replication and therefore used as a positive control in this study (Fig. 7A)^26^. 66E2 as well as CMC inhibited HEV replication close to 50% at 10 μM without showing any significant cytotoxicity (Fig. 7A and B). Furthermore, estimation of intracellular levels of HEV RNA by quantitative PCR analysis revealed that 66E2 and CMC inhibited HEV replication close to 65% and 95%, respectively, at 10 μM (Fig. 7C).

Next, the inhibitory potential of 66E2 was analyzed using infectious p6 virus. For this, S10-3 cells were electroporated with *in vitro* transcribed HEV p6 RNA. Media was collected at different time points and the presence of viral RNA analyzed using reverse transcriptase PCR. Maximum viral RNA was observed at 16 days post electroporation (Fig. S4). To determine whether the secreted virus particles are infectious, fresh S10-3 cells were incubated with 16-day virus containing media. After 6 days, compounds 66E2 and CMC along with DMSO control were added and cultures were incubated for further 48 hours. Total RNA was extracted and levels of viral sense and antisense RNA were analyzed by quantitative PCR (Fig. 7D and E). 66E2 showed more than 80% inhibition of both sense and antisense RNA of HEV similar to CMC. In agreement with the results obtained with the p6/luc replicon (Fig. 7A and C), 66E2 showed marked antiviral activity against infectious HEV. Lastly, an *in vitro* RdRp assay was conducted to evaluate the effect of 66E2 on HEV RdRp activity (Fig. 7F)^27^. Neither 66E2 nor CMC showed any significant inhibition of RdRp activity, suggesting that they did not act directly on the RdRp (Fig. 7F). Furthermore, quercetagetin, a potent inhibitor of HCV RdRp was able to inhibit HEV RdRp only marginally.

In summary, our data indicate that 66E2 could inhibit HCV, DENV and HEV and thus displays a broad-spectrum antiviral effect.

## Discussion

All known RNA viruses encode RNA-dependent RNA polymerases (RdRp), which interact with other viral and host factors to form a replicase complex responsible for their replication. RdRps have been an attractive antiviral target given their vital role in viral replication. The advantages of targeting RdRp include identification of a pan-genomic inhibitor and obtaining an inhibitor with higher barrier to resistance^28, 29^. In addition to viral proteins, targeting of host proteins provides another antiviral strategy with the added benefit of higher genetic barrier to resistance^30^. A host protein necessary for multiple viruses could be an attractive target for broad-spectrum antivirals.

With these objectives in mind we used the cell-based assay for HCV-3a RdRp for screening of small molecule inhibitors. Earlier, we have shown that HCV genotype 1b inhibitors, benzothiadiazine derivatives could inhibit NS5B activity using this assay^19^. This provided us the proof of principle that the cell-based assay could be used for screening of antivirals. Furthermore, being a cell-based assay it provided us the opportunity to identify antiviral molecules targeting the host protein(s) in addition to the RdRp.

Since HCV inhibitors against NS3, NS5A and NS5B have shown less efficiency against genotype 3 than other genotypes of HCV^31^, we screened for antiviral molecules with genotype 3a NS5B. Upon screening of NCI’s diversity set II library, we identified four compounds with potential anti-HCV activity (57G7, 59B9, 64C5 and 66E2). One of them, 57G7 was a RIG-I inhibitor and two others (59B9 and 64C5) did not show any inhibition in the HCV-3a replicon assay (Figs 1 and 2). The latter two compounds could inhibit HCV-3a RdRp activity in the cell-based assay but not in the replicon assay. This difference could be attributed to the fact that in the RdRp cell-based assay only HCV NS5B is overexpressed in the cells but in the replicon assay NS3-NS5B proteins are expressed from the replicon RNA. The presence of other HCV proteins could have caused hindrance to the interaction of 59B9 and 64C5 with their target site/proteins and preventing it from inhibiting replication of the RNA. This hypothesis was supported by the fact that at higher concentrations, these compounds could show inhibition of HCV replicon (Fig. S2). However, EC_50_ for 59B9 and 64C5 was more than 20 and 50 μM, respectively. Hence these two compounds were not considered for further analyses. Thus from a screen of 1363 compounds we obtained one HCV-3a inhibitor which showed significant inhibition of both positive and negative strand RNA as well as its protein expression. In addition, the compound inhibited RdRps from all six major genotypes of HCV indicating its pangenotypic potential (Fig. 4).

*In vitro* RdRp assay revealed that 66E2 was unable to inhibit recombinant HCV-3a NS5BΔ21 and is probably not a direct acting antiviral. While quercetagetin inhibited RdRp, CMC and sofosbuvir showed properties similar to that of 66E2 (Fig. 5B). On the other hand, 66E2 along with CMC and sofosbuvir showed very potent anti-HCV-3a property in replicon assay while quercetagetin was inert (Fig. 5C). Quercetagetin was shown to inhibit RNA binding to NS5B and was capable of efficiently inhibiting HCV genotypes 1b and 2a replication in cell culture^23^. However, in our hands quercetagetin did not inhibit HCV-3a replicon. It is possible that quercetagetin is not effective against genotype 3 HCV replicon at the concentration tested. Even though RdRp is the catalytic subunit responsible for the formation of phosphodiester bonds during viral RNA synthesis, other viral encoded and host proteins are involved in its functioning. Thus RNA synthesis could be abrogated if any one of the proteins in the multiprotein replicase complex is interfered with. The inability to inhibit recombinant NS5BΔ21 suggests that 66E2, like CMC and sofosbuvir might undergo metabolic changes within the cellular milieu or interact with a host protein necessary for viral replication. One of the host proteins that have been targeted for treatment of HCV infected patients is cyclophilin. HCV NS5A interacts with cyclophilin A leading to the formation of double membrane vesicles necessary for HCV replication^32, 33^. Furthermore cyclophilin inhibitors were shown to remodel endoplasmic reticulum in HCV-infected cells rendering them resistant to reinfection^34^.

CMC, a nucleoside analog inhibitor of HCV RdRp showed significant promise as a potential HCV therapeutic agent^35^. However, pharmacokinetic studies revealed that CMC suffered from low oral bioavailability^36^. Even the modified version of CMC, 3′-*O*-valinyl ester prodrug of 2′-*C*-methylcytidine with improved oral bioavailability had to be put on hold due to severe side effects^37^. In our hands, even though CMC was a better inhibitor of HCV-3a and HEV, it was quite inferior to 66E2 in inhibiting DENV (Figs 2, 6 and 7). Also, upon prolonged exposure to CMC, emergence of HCV NS5B mutant (S282T) resistant to CMC was reported^21^. Since 66E2 targets host protein(s), chances of developing drug resistant mutants are very low.

Limited structure-function analyses of 66E2 revealed that separating out the ethylcarbazol and the nitrophenylurea led to loss of antiviral activity (Fig. 3). This suggests that both moieties are necessary for its interaction with the host protein or for its further metabolic modification to generate the active form. Remarkably, 66E2 not only inhibited RdRps from all the six major genotypes of HCV but also inhibited replication of viruses such as DENV and HEV (Figs 6 and 7). These data further support the reasoning that 66E2 may be interacting with a host protein or undergoing conversion into an active form.

We note that 66E2 is capable of inhibiting viruses belonging to different families. While HEV is classified as a Hepevirus in the family Hepeviridae, HCV is a member of Hepacivirus genus in the family Flaviviridae. DENV also belongs to the family Flaviviridae but in the genus Flavivirus. The other members of the flaviviruses include, the yellow-fever virus, West Nile, Japanese encephalitis and Zika. It will be interesting to see if 66E2 would be able to inhibit these economically important viruses as well.

Due to the rapid mutating nature of RNA viral genome, the emergence of drug-resistant mutants has been a major issue for the DAA based treatment. Given the high cost and the time involved in drug development, the conventional one-bug-one-drug approach is inadequate to meet the threat of the emerging and re-emerging viruses^30^. Hence, the inhibitor reported in this study could be a starting point for new broad-spectrum antiviral drug discovery especially targeting host proteins.

## Methods

### Plasmids, viral RNA transcripts, cells

Plasmids used for expression of HCV NS5B from all six major genotypes were as reported earlier^19^. The cDNA of RIG-I (pUNO-hRIG) was from Invivogen (San Diego, CA). Huh7 and Huh7.5 cells were originally obtained from Dr. C.M. Rice. S10-3 cells are a subclone of Huh7 cells and were a kind gift from Dr. S. Emerson. The HEK 293T cells were from the ATCC (USA). Huh7, Huh7.5, S10-3 and HEK293T cells were maintained in Dulbeco’s modified Eagle medium (DMEM) containing 10% fetal calf serum, 50 I.U./ml Penicillin and Streptomycin in 5% CO_2_. All cells were grown at 37 °C except S10-3, which was incubated at 34.5 °C as reported^25^. Cells were transfected using Lipofectamine 2000 (Life Technologies, USA) as per the manufacturer’s protocol.

### Antibodies and reagents

Antibodies against HCV NS5A (ab13833) and DENV (ab26837 and ab9202) were from Abcam and anti GAPDH was purchased from Santa Cruz Biotechnology (USA). Compounds, 1-benzyl-3-(2-methyl-4-nitrophenyl)urea, 1,3-Bis(4-nitrophenyl)urea, 3-Amino-9-ethylcarbazole and 2′-*C*-Methylcytidine were purchased from Sigma (USA). Sofosbuvir was purchased from Selleckchem (Houston, TX).

### Cell-based assay

Cell-based assay was performed as reported earlier in CoStar White 96-well plates^19^. Briefly, plasmids expressing NS5B were co-transfected along with plasmids to express RIG-I, as well as two luciferases: the firefly luciferase driven from an interferon-β promoter and a *Renilla* luciferase driven from a thymidine kinase (TK) promoter. Compounds (10 μM) were added 4 h after plasmid transfection. All transfections were performed using Lipofectamine 2000 following manufacturer’s protocol. Luciferase activity was measured 16 h after addition of the compounds using DualGlo Luciferase assay system reagents (Promega) according to manufacturer’s protocol. Luminescence was detected using Synergy HT Multi-Mode microplate reader (Bio-Tek, USA). Ratio of firefly to *Renilla* luciferase values was converted to percentages and plotted using Microsoft Excel. Value obtained with DMSO was considered as 100%.

For RIG-I assay, cells were transfected with plasmids expressing RIG-I and the two luciferase reporters as mentioned above. 24 h after introduction of the plasmids, RIG-I agonist 3PdsR27 (a dsRNA of 27 base pairs with 5′ triphosphate, ref. 38) was transfected into the cells at a 50 nM. Luciferase activity was measured 16 h post transfection of RNA agonist as described above.

### Replicon Assay

HCV genotype 3a bicistronic replicon S52/SG-Feo (AII) was a kind gift from Dr. C.M. Rice. The plasmid was linearized with XbaI and *in vitro* transcribed using AmpliScribe T7-Flash transcription kit (Epicentre Biotechnologies Inc., USA). Template DNA was removed with RNase-free DNase followed by phenol/chloroform extraction and precipitation of RNA. RNA quality was assessed by agarose gel electrophoresis. HCV replicon RNA was electroporated into Huh7.5 cells in a 4 mm cuvette (200 volts, 950 μF capitance, ∞ resistance) using BioRad GenePulser Xcell. Transfected cells were suspended in culture medium and transferred to 10 cm diameter dishes. The replicon expressing cells were selected by the addition of G418 (0.5 mg/ml) 48 h post transfection. Two weeks later G418 resistant cells were used for antiviral assays.

HEV genotype 3 replicon (p6/luc) plasmid was a kind gift from Dr. S. Emerson. Plasmid was linearized with MluI and capped transcripts were generated using mMESSAGE mMACHINE Kit (Life Technologies, USA). Huh7 cells were electroporated with p6/luc RNA as above. Cells were maintained in T75 flask with replacement of the media every three days. Replication of HEV was monitored by measuring luciferase activity of the gaussia luciferase secreted into the medium.

For inhibitor analyses, HCV and HEV replicon expressing cells were seeded into 96 well plates and after 24 h, inhibitors were added at desired concentrations. DMSO was used as a vehicle control. Care was taken to ensure all wells contained same amount of DMSO (less than 1% final). Luciferase activity was measured 48 h after addition of compounds.

### Cell Viability Assay

Cytotoxicity analysis was performed using water-soluble tetrazolium (WST-1) reagent (Roche, USA) as per manufacturer’s protocol. For cell-based assays cytotoxicity assay was performed after 16 h of incubation and 48 h of incubation for replicon assays. The viability of the cells was checked using 1:10 dilution of WST-1 reagent to the complete media. Cells were incubated and analyzed at 1 h and 2 h duration. The absorbance was measured at 450 nm and 630 nm respectively. Difference between the two absorbance readings was plotted as percentage values with DMSO treated samples considered as 100%.

### Dengue Virus growth and infection

DENV-2 was grown in C6/36 cells. Infection was set up at 0.2 MOI for 2 h in incomplete medium at cell confluency of ~70%. Infection medium was replaced with 2% FBS containing medium and virus was harvested at day 5 post-infection. Virus titration was done by focus forming assays in Vero cells. Briefly 10 fold-serial dilutions of the virus (in MEM with 2% FBS) was added to semi-confluent Vero cells in 24 well plate for 2 h at 37 °C with gentle rocking. Following removal of media and washing, 1 ml of maintenance media containing 2% FBS was added to cells. Cells were fixed after 72 h with 2% paraformaldehyde followed by staining with D1-4G2-4-15 (ATCC HB-112) primary antibody and Alexa 488 anti-Mouse secondary antibody. No of foci were counted by under fluorescence microscope and used to calculate FFU/ml.

For DENV-2 infection, Huh 7 cells were infected by incubating DENV-2 virus at 1 MOI at 37 °C with gentle rocking. After 2 h cells were washed with PBS and fresh media along with compounds at indicated concentrations were added. Cells were lysed at 48 h post infection in Trizol reagent and processed for RNA isolation.

### Immunofluorescence

Huh-7 cells were either mock infected or infected with DENV 2 at 1MOI. At 48 h pi cells were fixed and stained with anti-dengue virus antibody (ab9202) and Alexa 488 anti-mouse antibody (green). Nuclei were stained with DAPI (blue). Cells were imaged under a 60× objective on FV1000 confocal microscope.

### ELISA

Huh7 cells were infected with DENV-2 as mentioned above. The cells were lysed at 48 h post infection using RIPA buffer. Anti-dengue rabbit polyclonal antibody, ab26837 was diluted in 50 mM carbonate-bicarbonate buffer (1:300) and coated onto F 96 Maxisorp flat-bottom ELISA plates (Nunc-immuno, Thermo Fischer) and incubated overnight at 4 °C. Two wells were kept as only buffer blank without the antibody. Wells were blocked with 5% BSA and treated with cell lysates for 2 h before adding the detection antibody (anti-dengue mouse monoclonal ab9202). Secondary antibody was an HRP conjugated anti-mouse antibody. Wells were washed with 1X PBS thrice between each antibody addition and five times before adding the substrate. 100 μl of TMB liquid substrate (Sigma Aldrich) was added to each well and the reaction was stopped after 15 minutes using equal volumes of 1 M H_2_SO_4_. Absorbance was measured at 450 and 630 nm and the difference between the two absorbance readings were plotted as percentage values with DMSO treated infected sample taken as 100%.

### Determination of DENV RNA copy number

The following primers specific for the DENV 3′ UTR were used for qPCR analysis^39^. Forward primer: 5′-GARAGACCAGAGATCCTGCTGTCT-3′.

Reverse primer: 5′-ACCATTCCATTTTCTGGCGTT-3′. A standard curve of log of copies vs Cq (quantitifaction cycle) values was generated using the DENV 3′ UTR clone. The exponential amplification value (calcuated as E_AMP_ = 10^(−1/m)^; m = slope) obtained was 2.099. The initial number of target DENV copies was obtained using the formula **X**_**o**_ **= E**_**AMP**_^**(b−Cq)**^ where **b** = y-intercept of the standard plot and **Cq** is the averaged observed Cq value of the amplification of target in each biological sample.

The PCR conditions were as follows: 94 °C for 2 min (1 cycle), 94 °C for 15 sec, 55 °C for 30 sec and 72 °C for 1 min (40 cycles). The qRT-PCR was done on Applied Biosystems ABI 7500 instrument.

### Western blotting

Huh7.5 cells expressing HCV-3a replicon was treated with indicated concentrations of 66E2 and 2′-*C*-Methylcytidine (CMC). 48 h post addition of the compounds cells was lysed with SDS-loading dye. Western blot was performed using antibodies against HCV NS5A and GAPDH using standard protocol.

### RNA isolation and reverse transcriptase quantitative PCR

Total RNA was isolated using Trizol reagent (Life Technologies, USA) followed by reverse transcription using ImProm-II reverse transcription system (Promega). Random hexamers and specific tag primers were used for cDNA synthesis for detecting sense and antisense strands respectively. Quantitative PCR was performed as described^27^. Primer sequences are available upon request.

### RdRp activity assay

The HCV genotype 3a NS5B protein lacking the C-terminal 21 amino acids (NS5B Δ21) containing a C-terminal hexa-histidine tag was expressed and purified from *E*. *coli* by using Talon metal affinity resin (Clontech) as described earlier^40^. The HCV RdRp assay consisted of 1 pmole of RNA template, LE21 and 0.04 mM of recombinant HCV polymerase in a 20 μL reaction^40^. The final buffer contained 20 mM sodium glutamate (pH 8.2), 20 mM NaCl, 4 mM MgCl_2_, 12.5 mM dithiothreitol, 0.5% (v/v) Triton X-100, 200 mM GTP, and 100 mM each of CTP and UTP, and 250 nM α-[P^32^]-ATP. The reactions were incubated at 30 °C for 60 min. The products were separated by electrophoresis on denaturing (7.5 M urea) polyacrylamide gels. The gels were wrapped in plastic, and radiolabel was quantified using a PhosphorImager.

HEV RdRp assay was performed as described earlier^27^.

### Data availability statement

All data generated or analyzed during this study are included in this published article (and its Supplementary Information Files).

## Electronic supplementary material

Supplementary Material
