# Supplementary material for: A screen for novel hepatitis C virus RdRp inhibitor identifies a broad-spectrum antiviral compound
Source: Sci Rep. 2017 Jul 19;7:5816. doi: 10.1038/s41598-017-04449-3 (PMC5517564; doi:10.1038/s41598-017-04449-3)
Supplement: Supplementary file 1 — Supplementary Material [file 41598_2017_4449_MOESM1_ESM.pdf]

A screen for novel hepatitis C virus RdRp inhibitor identifies a broad-spectrum antiviral compound

Abhilasha Madhvi[a#], Smita Hingane[a#], Rajpal Srivastav[a], Nishant Joshi[a,b], Chandru Subramani[a], Rajagopalan Muthumohan[a], Renu Khasa[a], Shweta Varshney[a], Manjula Kalia[a], Sudhanshu Vrati[a,c], Milan Surjit[a] and CT Ranjith-Kumar[a*]

Vaccine and Infectious Disease Research Center, Translational Health Science and Technology Institute, Faridabad, India[a].

Present Address: Shiv Nadar University, Gautam Buddha Nagar, Uttar Pradesh, India[b]; Regional Centre for Biotechnology, Faridabad, India[c].

*Address correspondence to CT Ranjith-Kumar, ctrkumar@thsti.res.in

# A.M. and S.H. contributed equally to this work

Supplemental Figures and Legends

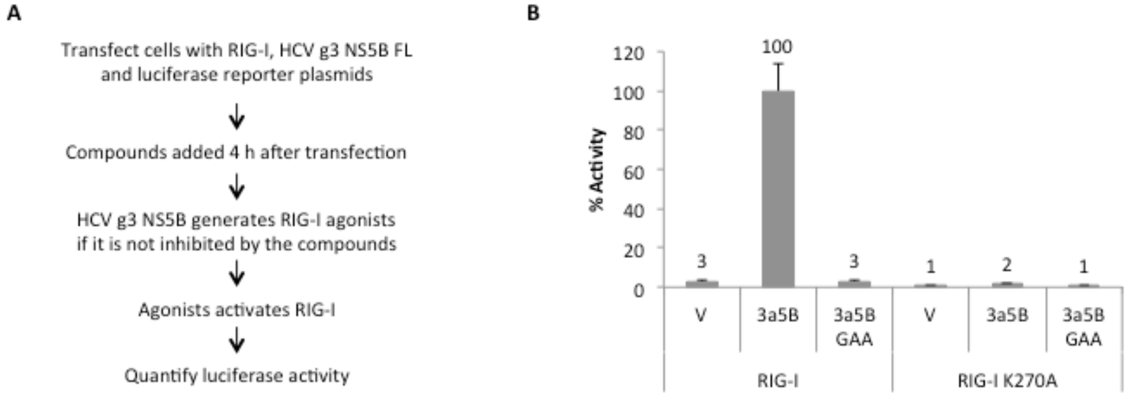

Supplemental Fig. 1 Cell-based assay for HCV genotype 3a NS5B. A) Flow chart depicting how the cell-based assay was performed. B) Characterization of HCV-3a RdRp using the cell-based assay. The mean % of the ratio of firefly luciferase to Renilla luciferase (referred to as % Activity) is plotted against ectopically expressed RdRp and its mutant. Wild type NS5B (3a5B) expressing cells induced RIG-I signaling. The control cells expressing vector alone (V) or the catalytic mutant (3a5B GAA, divalent metal binding motifs of GDD was mutated to GAA) was inactive demonstrating that RNA synthesis by NS5B was required for RIG-I activation. Also mutant RIG-I (K270A, with a substitution at the ATP binding domain) failed to be activated by NS5B. The catalytically active NS5B and signaling competent RIG-I was necessary for the assay suggesting that the assay is dependent on catalytically active HCV-3a NS5B. The assay was performed in triplicates and results presented are representative of three independent assays.

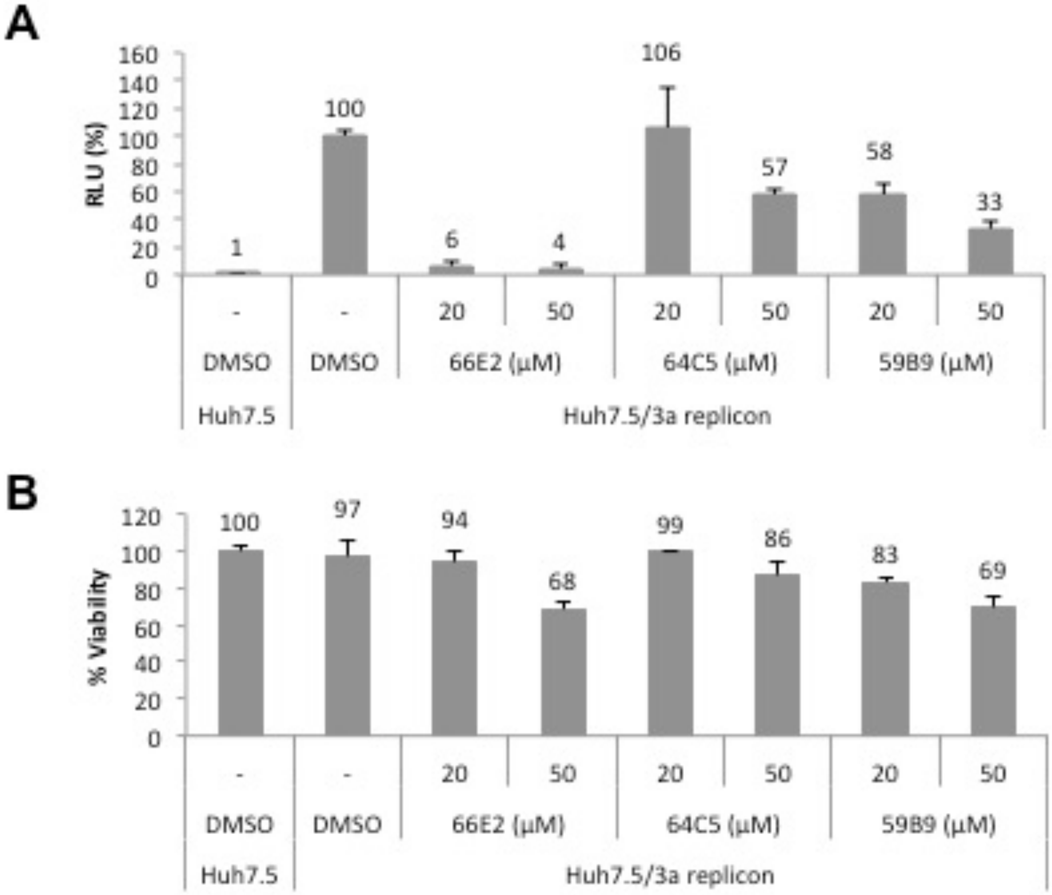

Supplemental Fig. 2. Assays with HCV genotype 3a replicon with higher concentrations of the compounds. A) G418 resistant HCV-3a replicon expressing Huh7.5 cells were treated with indicated compounds at 20 and 50 µM for 48 h and the firefly luciferase activity was plotted as relative luciferase units (RLU). DMSO treated HCV-3a replicon expressing Huh7.5 cells was taken as 100%. B) The toxicity of these compounds in the replicon expressing cells was measured using WST-1 assay reagent. The values are depicted as percentages with the DMSO treated cells taken as 100%.

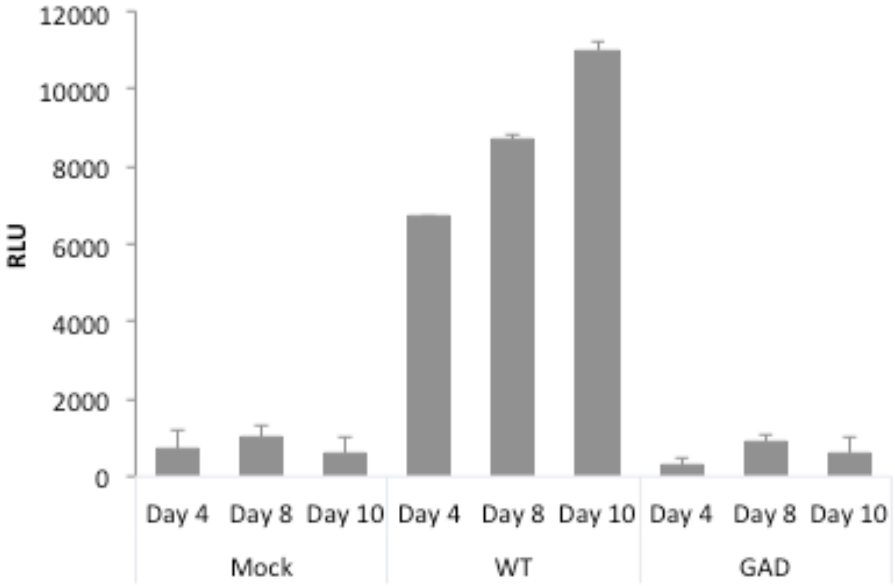

Supplemental Fig. 3. Characterization of HEV p6/luc reporter system. Huh7 cells were electroporated with mock, capped p6/luc wild type (WT) and mutant (GAD, divalent metal binding motifs of GDD was mutated to GAD) transcripts. Gaussia luciferase secreted into the media was measured at different time points after electroporation as mentioned on the x-axis. GAD mutant did not show any luciferase activity suggesting that the observed activity is due to RNA replication.

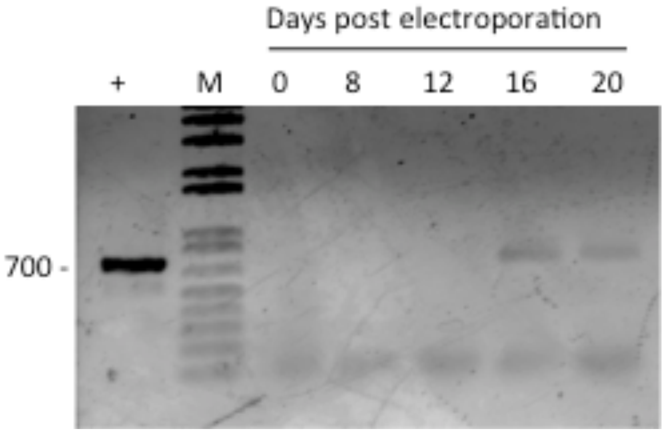

Supplemental Fig. 4. Secretion of virus particles from S10-3 cells. S10-3 cells were electroporated with in vitro transcribed capped HEV p6 RNA transcripts. Media was collected at different time points after electroporation. Total RNA was extracted from 500µl of media and cDNA synthesized using random primers. PCR was performed with primers corresponding to Y domain of ORF1. In vitro transcribed RNA was used as positive control (+). M corresponds to DNA ladder and the numbers correspond to the days in which media was collected post electroporation of the RNA.

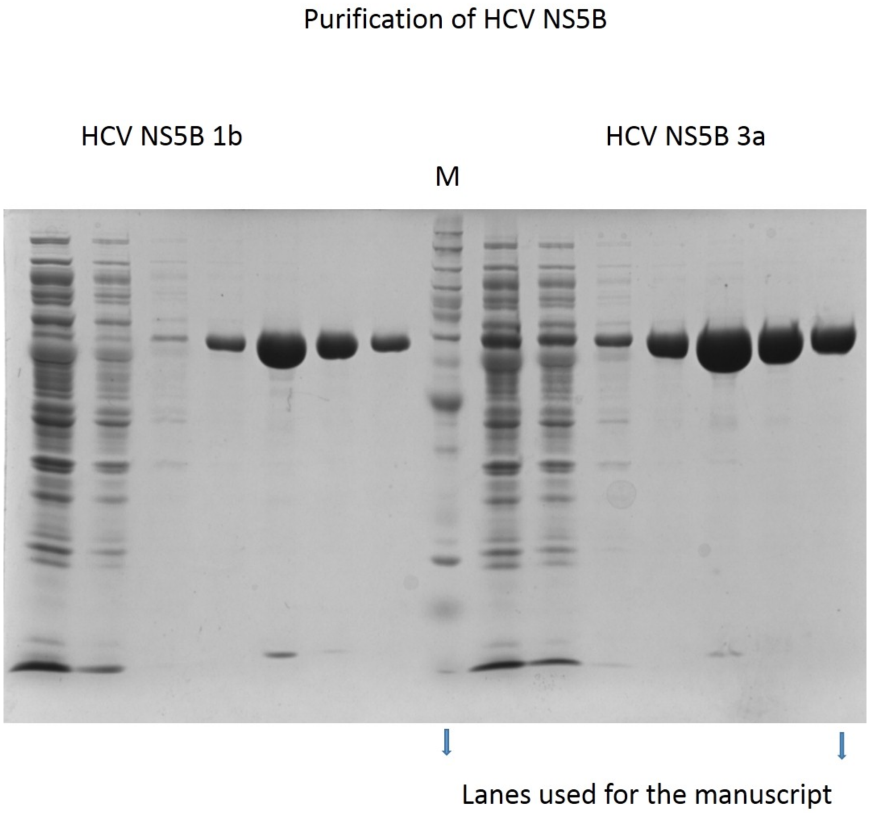

Supplemental Fig. 5. Purification of HCV NS5B (RdRp) lacking C-terminal 21 amino acids from genotype 1b and 3a (HCV NS5B 1b and HCV NS5B 3a). The purification was performed as described in the Methods section. The entire coomassie stained SDS-PAGE used for generating Fig. 5A is shown. The lanes used for the figure 5A are indicated below the gel.
